# Supplementary material for: Cortical state transitions and stimulus response evolve along stiff and sloppy parameter dimensions, respectively
Source: eLife. 2020 Mar 17;9:e53268. doi: 10.7554/eLife.53268 (PMC7108864; doi:10.7554/eLife.53268)
Supplement: Transparent reporting form [file elife-53268-transrepform.docx]

***eLife’s* transparent reporting form**

We encourage authors to provide detailed information *within their submission* to facilitate the interpretation and replication of experiments. Authors can upload supporting documentation to indicate the use of appropriate reporting guidelines for health-related research (see [EQUATOR Network](http://www.equator-network.org/%20)), life science research (see the [BioSharing Information Resource](https://biosharing.org/)), or the [ARRIVE guidelines](http://www.plosbiology.org/article/info:doi/10.1371/journal.pbio.1000412) for reporting work involving animal research. Where applicable, authors should refer to any relevant reporting standards documents in this form.

If you have any questions, please consult our Journal Policies and/or contact us: [editorial@elifesciences.org](mailto:editorial@elifesciences.org).

**Sample-size estimation**

- You should state whether an appropriate sample size was computed when the study was being designed
- You should state the statistical method of sample size computation and any required assumptions
- If no explicit power analysis was used, you should describe how you decided what sample (replicate) size (number) to use

Please outline where this information can be found within the submission (e.g., sections or figure legends), or explain why this information doesn’t apply to your submission:

No explicit power analysis was used to calculate sample size. The sample size (n = 6) was a trade – off between reaching a statistical power and minimizing number of animals used for experiments. We have followed the standards accepted in the field where sample size between 5 and 10 rats is a common practice.

**Replicates**

- You should report how often each experiment was performed
- You should include a definition of biological versus technical replication
- The data obtained should be provided and sufficient information should be provided to indicate the number of independent biological and/or technical replicates
- If you encountered any outliers, you should describe how these were handled
- Criteria for exclusion/inclusion of data should be clearly stated
- High-throughput sequence data should be uploaded before submission, with a private link for reviewers provided (these are available from both GEO and ArrayExpress)

Please outline where this information can be found within the submission (e.g., sections or figure legends), or explain why this information doesn’t apply to your submission:

The number of the animals used for experiments is stated in the “Experimental Techniques” subsection of the “Methods” and repeated in the “Result” section. We did not exclude outliers. The range of the number of analyzed neurons is indicated in the “Result” section. A detailed data description (e.g. number of single units, number of stimulus presentation) is stated in the Table1. The surrogate data sets had the same statistic as a corresponding experimental data set. Further experimental procedures (surgery) and spikes sorting procedures have been previously described in Mochol et al. (2015, PNAS) (https://doi.org/10.1073/pnas.1410509112)

**Statistical reporting**

- Statistical analysis methods should be described and justified
- Raw data should be presented in figures whenever informative to do so (typically when N per group is less than 10)
- For each experiment, you should identify the statistical tests used, exact values of N, definitions of center, methods of multiple test correction, and dispersion and precision measures (e.g., mean, median, SD, SEM, confidence intervals; and, for the major substantive results, a measure of effect size (e.g., Pearson's r, Cohen's d)
- Report exact p-values wherever possible alongside the summary statistics and 95% confidence intervals. These should be reported for all key questions and not only when the p-value is less than 0.05.

Please outline where this information can be found within the submission (e.g., sections or figure legends), or explain why this information doesn’t apply to your submission:

Whenever possible, raw data points were presented as scatter plots (Fig. 2A; Fig. 5B,E; Fig. 6D) and data for each animal were presented in separated panels (Fig. 4E; Fig. 6D).

All error bars were defined in their respective figure legend.

The p-values of correlations and statistical tests were reported in the figure legends. p-values between 0.001 and 0.05 were written explicitly, p-values lower than were reported as "p < 0.001" or through an asterisk, and p-values larger than 0.05 were noted "n.s.". For results not presented in the main figures, statistical tests and p-values were reported in the results section of the text.

(For large datasets, or papers with a very large number of statistical tests, you may upload a single table file with tests, Ns, etc., with reference to sections in the manuscript.)

**Group allocation**

- Indicate how samples were allocated into experimental groups (in the case of clinical studies, please specify allocation to treatment method); if randomization was used, please also state if restricted randomization was applied
- Indicate if masking was used during group allocation, data collection and/or data analysis

Please outline where this information can be found within the submission (e.g., sections or figure legends), or explain why this information doesn’t apply to your submission:

We did not divide the animals into different groups.

**Additional data files (“source data”)**

- We encourage you to upload relevant additional data files, such as numerical data that are represented as a graph in a figure, or as a summary table
- Where provided, these should be in the most useful format, and they can be uploaded as “Source data” files linked to a main figure or table
- Include model definition files including the full list of parameters used
- Include code used for data analysis (e.g., R, MatLab)
- Avoid stating that data files are “available upon request”

Please indicate the figures or tables for which source data files have been provided:

We made the spiking data publicly available here:

https://github.com/adrianponce/Spont_stim_spiking_A1
